# Cryptic β-Lactamase Evolution Is Driven by Low β-Lactam Concentrations

**DOI:** 10.1128/mSphere.00108-21

**Published:** 2021-04-28

**Authors:** Christopher Fröhlich, João A. Gama, Klaus Harms, Viivi H. A. Hirvonen, Bjarte A. Lund, Marc W. van der Kamp, Pål J. Johnsen, Ørjan Samuelsen, Hanna-Kirsti S. Leiros

**Affiliations:** a The Norwegian Structural Biology Centre (NorStruct), Department of Chemistry, UiT The Arctic University of Norway, Tromsø, Norway; b Department of Pharmacy, UiT The Arctic University of Norway, Tromsø, Norway; c School of Biochemistry, University of Bristol, Bristol, United Kingdom; d Hylleraas Centre for Quantum Molecular Sciences, Department of Chemistry, UiT The Arctic University of Norway, Tromsø, Norway; e Norwegian National Advisory Unit on Detection of Antimicrobial Resistance, Department of Microbiology and Infection Control, University Hospital of North Norway, Tromsø, Norway; Antimicrobial Development Specialists, LLC

**Keywords:** OXA-48, ceftazidime, resistance development, cryptic evolution, *Escherichia coli*, carbapenemase, carbapenem, sub-MIC, structural flexibility, catalytic efficiency

## Abstract

Our current understanding of how low antibiotic concentrations shape the evolution of contemporary β-lactamases is limited. Using the widespread carbapenemase OXA-48, we tested the long-standing hypothesis that selective compartments with low antibiotic concentrations cause standing genetic diversity that could act as a gateway to developing clinical resistance. Here, we subjected Escherichia coli expressing *bla*_OXA-48_, on a clinical plasmid, to experimental evolution at sub-MICs of ceftazidime. We identified and characterized seven single variants of OXA-48. Susceptibility profiles and dose-response curves showed that they increased resistance only marginally. However, in competition experiments at sub-MICs of ceftazidime, they demonstrated strong selectable fitness benefits. Increased resistance was also reflected in elevated catalytic efficiencies toward ceftazidime. These changes are likely caused by enhanced flexibility of the Ω- and β5-β6 loops and fine-tuning of preexisting active site residues. In conclusion, low-level concentrations of β-lactams can drive the evolution of β-lactamases through cryptic phenotypes which may act as stepping-stones toward clinical resistance.

**IMPORTANCE** Very low antibiotic concentrations have been shown to drive the evolution of antimicrobial resistance. While substantial progress has been made to understand the driving role of low concentrations during resistance development for different antimicrobial classes, the importance of β-lactams, the most commonly used antibiotics, is still poorly studied. Here, we shed light on the evolutionary impact of low β-lactam concentrations on the widespread β-lactamase OXA-48. Our data indicate that the exposure to β-lactams at very low concentrations enhances β-lactamase diversity and drives the evolution of β-lactamases by significantly influencing their substrate specificity. Thus, in contrast to high concentrations, low levels of these drugs may substantially contribute to the diversification and divergent evolution of these enzymes, providing a standing genetic diversity that can be selected and mobilized when antibiotic pressure increases.

## Abstract (precis)

Very low antibiotic concentrations have been shown to drive the evolution of antimicrobial resistance. While substantial progress has been made to understand the driving role of low concentrations during resistance development for different antimicrobial classes, the importance of β-lactams, the most commonly used antibiotics, is still poorly studied.

## INTRODUCTION

Since the discovery of the first β-lactam, penicillin, this antimicrobial class has diversified into a broad range of agents, and it remains the most widely used class of antimicrobials worldwide (1). The extensive use of these agents has inevitably led to the selection of multiple resistance mechanisms where the expression of β-lactamase enzymes plays a major role, particularly in Gram-negative bacteria (2). Consequently, β-lactamases are arguably among the most-studied enzymes worldwide. Considerable progress has been made in understanding their molecular epidemiology and biochemical properties (3, 4). The evolutionary forces driving the diversification of these enzymes are, however, poorly understood. Already, more than 20 years ago, it was proposed that suboptimal antibiotic concentrations within the host fuel the evolution of β-lactamases, altering their substrate profiles (5–8). This “compartment hypothesis” was later supported by a series of studies unequivocally demonstrating that selection for antibiotic resistance determinants can occur at very low antibiotic concentrations (9–11). These low concentrations are thus widening the window in which selection can take place (12). Despite their clinical significance, few studies have investigated the effects of sub-MICs of β-lactams on the evolution and selection of contemporary, globally circulating β-lactamases (5, 13, 14).

Within the last decade, OXA-48 has become one of the most widespread serine β-lactamases. This Ambler class D β-lactamase confers resistance toward penicillins and decreases susceptibility to our last-resort drugs, the carbapenems. However, it is a poor hydrolyzer of extended-spectrum cephalosporins, including ceftazidime (15–17). Despite that, naturally occurring OXA-48-like variants have been identified exhibiting increased ceftazidime activity but antagonistic pleiotropy toward penicillins and carbapenems (e.g., OXA-163, OXA-247, and OXA-405) (18–20). Ceftazidime resistance development in these variants was mostly due to single amino acid changes and a shortened β5-β6 loop (4, 18–21). We previously showed that exposure to increasing concentrations of ceftazidime can select for this latent ceftazidimase function of OXA-48 in the laboratory (17).

To test the long-standing hypothesis, that β-lactams at sub-MICs can drive the evolution of these enzymes, we subjected Escherichia coli MG1655 expressing OXA-48 to concentrations of ceftazidime below the MIC (0.25× MIC). Over the course of 300 generations, we identified seven single variants of OXA-48 (L67F, P68S, F72L, F156C/V, L158P, and G160C). Their ceftazidime MICs were indistinguishable or only marginally increased compared to wild-type OXA-48. However, when expressed at sub-MICs of ceftazidime, all allele variants conferred strong fitness benefits. Measuring dose-response curves (50% inhibitory concentration [IC_50_]) and enzyme kinetics revealed further that (i) all genotypes decreased ceftazidime susceptibility significantly and (ii) all enzyme variants exhibited increased catalytic efficiencies against ceftazidime. Molecular dynamics (MD) simulations of P68S, F72L, and L158P showed elevated flexibility of both the Ω (D143 to I164) and β5-β6 (T213 to K218) loops likely to aid hydrolysis of the bulkier ceftazidime by increasing active site accessibility. Structural investigations of L67F also revealed a novel binding pocket outside of the active site where the β5-β6 loop was involved in stabilizing the binding of the hydrolyzed ceftazidime molecule. Worryingly, double mutants, such as F72I/G131S (OXA-D320, GenBank accession no. KJ620465) and N146S/L158P (OXA-D319, GenBank accession no. KJ620462), were recently identified in environmental samples (22, 23), underlining the importance and evolutionary power of environments with low selective pressure.

## RESULTS

### Sub-MICs of ceftazidime select for high-level resistance.

**FIG 1:**
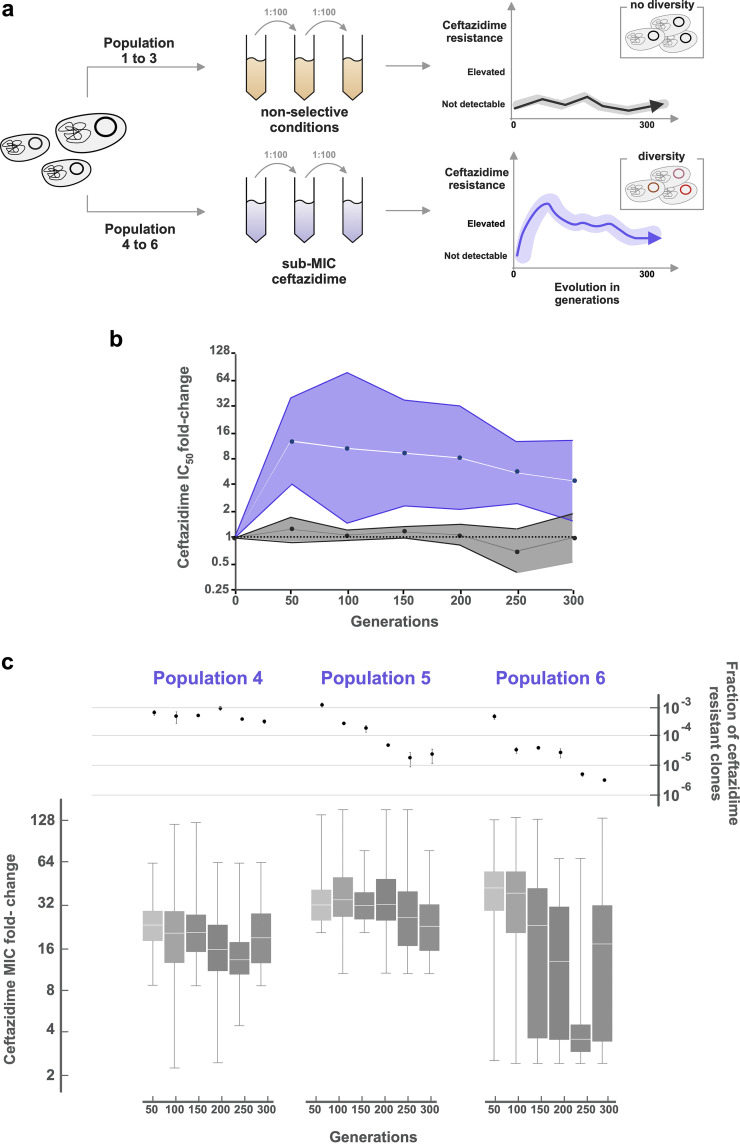
Population-level effects of sub-MIC ceftazidime exposure. (a) Experimental design. (b) IC_50_ fold change for populations evolved without (gray) and under sub-MIC ceftazidime conditions (violet), relative to wild-type OXA-48. Bands represent the standard deviation around the geometric mean. (c) The top section shows the fraction of clones able to grow on ceftazidime, 1 mg/liter (>2-fold MIC); the bottom section displays MIC fold change distributions of preselected clones. Box plots represent quartiles and the median of the distributions.

Here, we wanted to study the evolvability of the carbapenemase OXA-48 under sub-MICs of the cephalosporin ceftazidime. OXA-48 does not hydrolyze ceftazidime efficiently (19). However, we recently showed that the exposure to increasing concentrations of ceftazidime can select for OXA-48 variants with elevated activity toward ceftazidime (17). We used the previously constructed E. coli MG1655 (Table S1, MP13-06) (17) carrying a globally disseminated IncL plasmid with *bla*_OXA-48_ as the only antibiotic resistance gene. MP13-06 was evolved without selection pressure and at one quarter of the ceftazidime MIC (0.06 mg/liter), resulting in the populations 1 to 3 (Pop1 to 3) and 4 to 6 (Pop4 to 6), respectively (Fig. 1a).

10.1128/mSphere.00108-21.1 TABLE S1 Strains used and constructed in this study Table S1, DOCX file, 0.04 MB. Copyright © 2021 Fröhlich et al. 2021 Fröhlich et al. https://creativecommons.org/licenses/by/4.0/ This content is distributed under the terms of the Creative Commons Attribution 4.0 International license.

To elucidate the effect of ceftazidime, we measured dose-response curves of the whole evolved populations and calculated the ceftazidime concentrations inhibiting 50% of cell growth (IC_50_). In Pop1 to 3, evolution without selection pressure did not result in altered ceftazidime susceptibility (Fig. 1b). In contrast, under ceftazidime selection (Pop4 to 6), susceptibility decreased on average 16-fold already after 50 generations (Fig. 1b). We observed that, during the course of experimental evolution, the susceptibilities of Pop4 to 6 shifted toward lower ceftazidime resistance (Fig. 1b).

From the evolved populations, we measured the fraction of clones exhibiting a clinically significant MIC change (>2-fold) by nonselective and selective plating on 1 mg/liter ceftazidime. No clones were identified during selection-free evolution above the detection limit (10^−7^ of the population). Under sub-MIC conditions, we found a significant fraction of the populations to be able to grow on ceftazidime containing plates (Fig. 1c). While this fraction was stably maintained in Pop4, we found that Pop5 and Pop6 showed a significant reduction over time (Pearson correlation, *P* = 0.54, *P* = 0.01, *P* = 0.03).

To determine the MIC distribution of clones with increased MICs, we selected approximately 50 colonies every 50th generation from the selective plates and tested their susceptibility to ceftazidime (Fig. 1c). All preselected clones displayed a MIC increase ranging from 2- to 128-fold. For Pop 4 to 6, we found that on average, 11%, 28%, and 34% of the tested colonies exhibited MIC values above the clinical resistance breakpoint of 4 mg/liter, respectively (EUCAST breakpoint table v. 10.0). These results are consistent with recent reports demonstrating that low-level concentrations of antibiotics facilitate the selection of high-level resistance (9, 11).

### Sub-MIC evolution selects for beneficial single point mutations in *bla*_OXA-48_.

**FIG 2:**
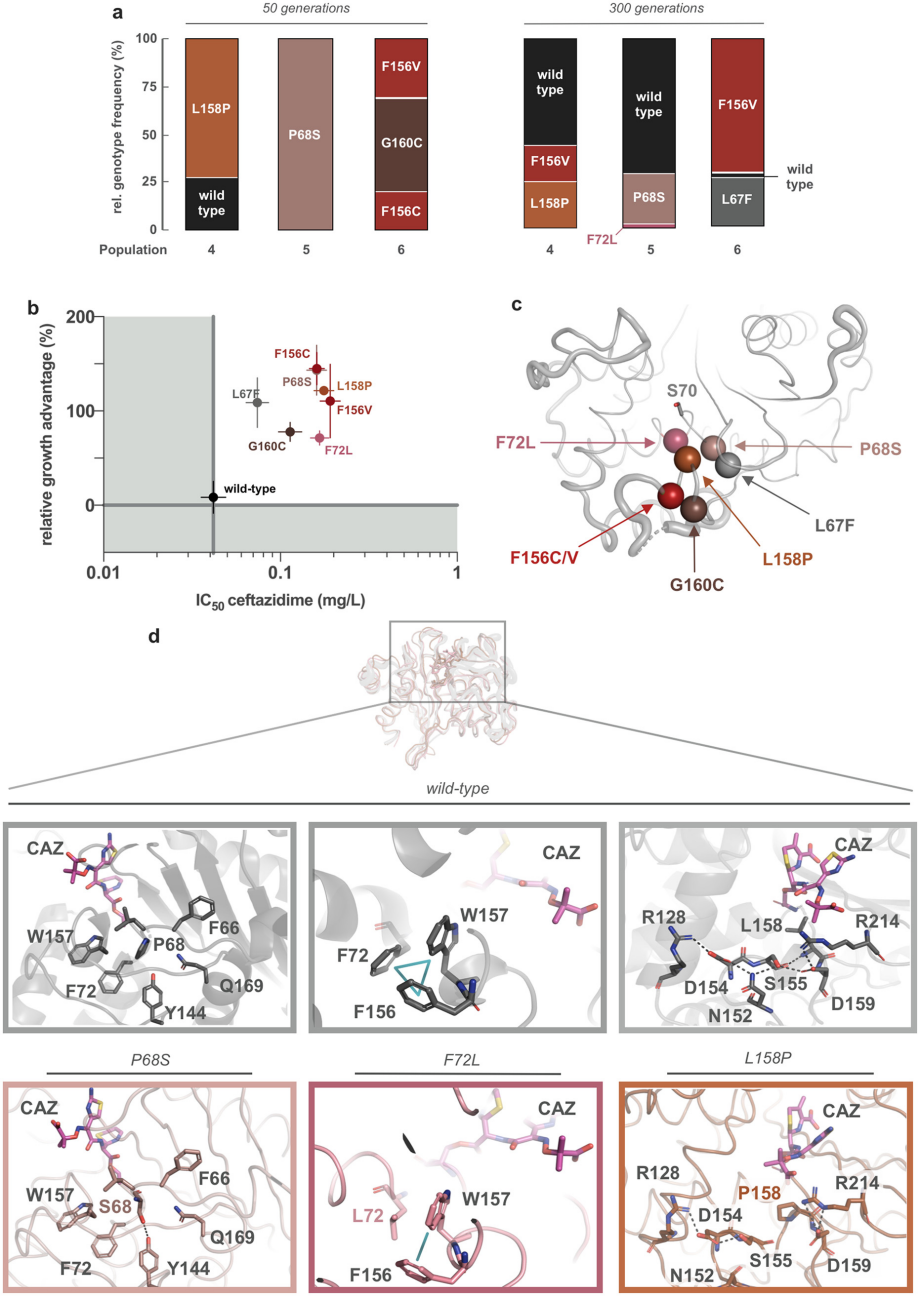
Phenotypic and structural investigation of OXA-48 allele variants. (a) Relative genotype frequencies of *bla*_OXA-48_ variants within Pop4 to 6, at 50 and 300 generations. (b) Relative growth advantage of OXA-48 variants expressed at sub-MICs of ceftazidime versus their ceftazidime IC_50_. Despite marginal changes in their ceftazidime susceptibility (IC_50_ increased by 2- to 4-fold), the expression of these alleles displays large fitness benefits at sub-MIC ceftazidime. Error bars represent the standard deviation. (c) Ribbon structure of OXA-48 including the amino acid changes close to the active site. (d) Representative structures from molecular dynamics simulations of wild type, P68S, F72L, and L158P performed with ceftazidime covalently bound to the active site S70. In short, S68 in P68S displays an H-bond with the tyrosine in the conserved Y^144^GN motif of OXA-48. F72L lacks the aromatic stacking interaction between F72 and F156/W157. L158P disrupts the H-bond network within the Ω-loop.

To understand the effect of sub-MIC exposure on OXA-48, we sequenced the *bla*_OXA-48_ gene of approximately 50 clones after 50 and 300 generations, which were preselected on agar plates containing 1 mg/liter ceftazidime. In total, seven single variants of OXA-48 were identified: L67F, P68S, F72L, F156C, F156V, L158P, and G160C. The relative frequency of these variants varied among populations and generations (Fig. 2a). Interestingly, double mutants with similar (F72I/G131S) or identical (N146S/L158P) amino acid changes have been already reported in environmental samples (23). To elucidate the effect of these second mutations, we constructed the OXA-48 double mutants F72L/G131S (instead of F72I) and N146S/L158P and included them in the following characterization.

To isolate the effects of OXA-48 on antimicrobial susceptibility, we subcloned all allele variants into a high copy number vector (pCR-Blunt II-TOPO) and expressed them in E. coli TOP10 (Table 1). As previously shown (17), the expression of OXA-48 resulted in up to 32- and 64-fold increased MIC toward penicillins and carbapenems (except for doripenem), respectively. While wild-type OXA-48 expression did not increase the MIC against cephalosporins (<2-fold), we found that P68S, F72L, L158P, and N146S/L158P resulted in 4- to 16-fold increased MIC against ceftazidime. Interestingly, the expression of all other alleles (L67F, F156C/V, G160C, F72L/G131S) did not increase the ceftazidime MIC significantly (i.e., not more than 2-fold compared to wild-type OXA-48). In addition, none of the alleles showed a significant effect on cephalosporins other than ceftazidime (Table 1). In contrast, the susceptibility to carbapenems and penicillins was increased by 2- to ≥64-fold for all the variants. The expression of F156C and G160C did not increase the MIC to any β-lactam. In clinical strains, *bla*_OXA-48_ is frequently located on IncL plasmids, which are typically present in low copy numbers (24). To mimic this situation, we subcloned all OXA-48 alleles into a low copy number vector (p15A origin, here called pUN), expressed these in E. coli MG1655, and repeated the ceftazidime MIC measurements. Within this more realistic genetic architecture, only F156V increased the ceftazidime MIC by more than 2-fold (Table 2).

**TABLE 1:** MIC of OXA-48 and allele variants expressed in the high copy number vector pCR-Blunt II-TOPO in E. coli TOP10

Antimicrobial agent(s)^*a*^	MICs (mg/liter) for:										
	MP13-04	MP13-11 wild-type OXA-48	MP13-21 L67F	MP13-16 P68S	MP13-14 F72L	MP13-17 F156C	MP13-18 F156V	MP13-15 L158P	MP13-19 G160C	MP13-33 F72L/G131S	MP13-20 N146S/L158P
Temocillin	16	256	64	64	64	16	16	64	16	16	32
Piperacillin-tazobactam	2	64	2	2	2	2	2	2	2	2	2
Amoxicillin-clavulanic acid	4	128	128	64	64	16	64	16	8	8	8
Ceftazidime	0.5	0.5	1	8	4	0.5	0.5	8	0.5	0.5	2
Ceftazidime-avibactam	0.25	0.25	0.5	0.25	0.5	0.5	0.5	0.5	0.5	0.25	0.5
Cefuroxime	16	16	8	16	8	8	8	8	8	16	8
Cefepime	0.06	0.12	0.06	0.12	0.12	0.06	0.06	0.25	0.06	0.06	0.12
Cefotaxime	0.12	0.25	0.12	0.12	0.12	0.12	0.12	0.25	0.12	0.25	0.12
Meropenem	0.03	0.25	0.12	0.03	0.03	0.03	0.03	0.03	0.03	0.03	0.03
Imipenem	0.25	1	0.25	0.25	0.25	0.25	0.25	0.25	0.25	0.25	0.5
Ertapenem	0.015	1	0.12	0.03	0.03	0.015	0.015	0.06	0.015	0.015	0.015
Doripenem	0.03	0.03	0.03	0.03	0.06	0.03	0.03	0.06	0.03	0.06	0.06

a Tazobactam fixed at 4 μg/ml; clavulanic acid fixed at 2 μg/ml; avibactam fixed at 4 μg/ml.

**TABLE 2:** Ceftazidime susceptibility (MIC and IC_50_) measurements of OXA-48 and variants expressed from the low copy number vector pUN in E. coli MG1655 *ΔmalF* (MP14-23)^*a*^

Measurement	Data for:										
	MP14-23	MP14-24 wild-type OXA-48	MP14-29 L67F	MP14-26 P68S	MP14-27 F72L	MP14-30 F156C	MP14-31 F156V	MP14-25 L158P	MP14-28 G160C	MP14-32 F72L/G131S	MP14-33 N146S/L158P
MIC (mg/liter)	0.5	0.5	0.5	1	1	1	2	1	0.5	0.5	1
IC_50_ (mg/liter)	0.045	0.053	0.074	0.160	0.167	0.161	0.191	0.176	0.114	0.078	0.220
CI95%	0.033–0.061	0.039–0.070	0.057–0.096	0.128–0.201	0.136–0.205	0.135–0.192	0.153–0.239	0.142–0.219	0.087–0.148	0.062–0.097	0.164–0.295

a Susceptibility was determined based on a minimum of 2 biological replicates. The 95% confidence intervals (CI95%) were calculated for the IC_50_ values.

Clinically insignificant or marginal changes in MICs have been reported to still confer high fitness benefits in the presence of low-level selection (5). To address this, we first increased the resolution of the susceptibility testing by measuring the dose-response curves in the low copy number vector. Calculating their corresponding IC_50_ values, we found that all variants conferred marginal but significant decreases in ceftazidime susceptibility (Fig. 2b and Table 2; analysis of variance [ANOVA], df = 10, *P* < 0.0001, followed by a Dunnett *post hoc* test with OXA-48 as the control group).

**FIG 3:**
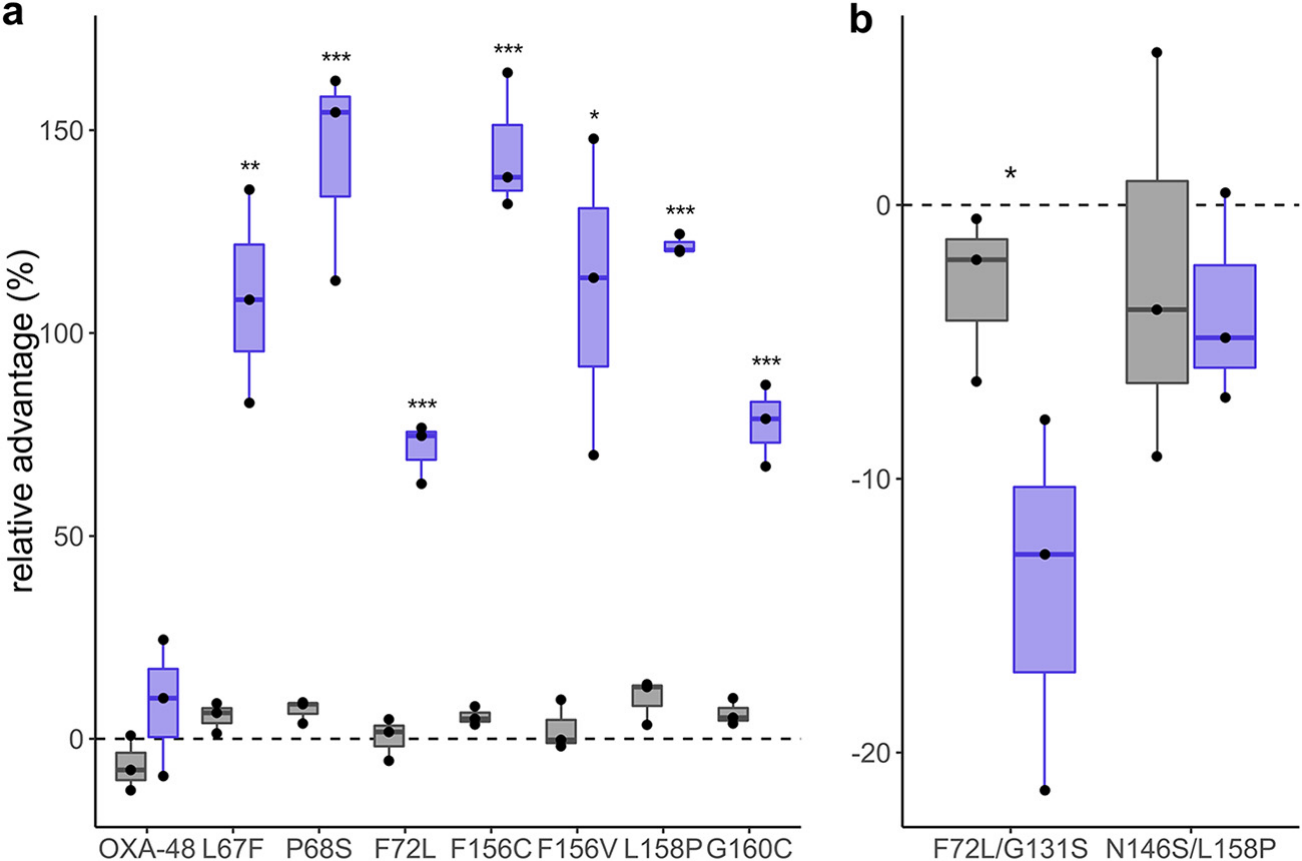
Head-to-head competitions. (a) E. coli MG1655 *mal^+^* competed with MG1655 *ΔmalF* expressing wild-type and allele variants of OXA-48, respectively, without (gray) and at sub-MICs (violet) of ceftazidime. While expression without selection pressure was neutral for all alleles, at sub-MICs, all allele variants showed fitness benefits over the wild-type allele. (b) E. coli MG1655 *mal^+^* expressing F72L versus *ΔmalF* F72L/G131S and *mal^+^* L158P versus *ΔmalF* N146S/L158P. G131S and N146S did not improve bacterial fitness at sub-MIC ceftazidime. The dots represent biological replicates, and significantly different averages compared to OXA-48 in the presence of ceftazidime (0.06 mg/liter) are marked with * (*P* < 0.05), ** (*P* < 0.01), and *** (*P* < 0.001).

Second, we performed head-to-head competitions between isogenic E. coli MG1655 strains (only differing in *ΔmalF*) to test the fitness effect of OXA-48 variants in the absence and presence of sub-MIC ceftazidime. To exclude an effect of the *malF* deletion on the bacterial fitness, we initially competed the strains both carrying the pUN vector encoding wild-type *bla*_OXA-48_ (MP08-61 and MP14-24). No significant change in bacterial fitness was observed in either condition (Welch *t*-test, *P* = 0.24 and *P* = 0.48), ruling out a detectable effect of the *malF* deletion. Next, we expressed all OXA-48 variants in MP14-23 and subjected those to competitions against MP08-61. Without ceftazidime, no difference in fitness was observed between variants and wild type (Fig. 3a; ANOVA, not assuming equal variances, df = 7, *P* = 0.33). However, at sub-MIC ceftazidime, all allele variants showed strong significant growth benefits (Fig. 2b and 3a; ANOVA, not assuming equal variances, df = 7, *P* = 0.0003, followed by a Dunnett *post hoc* test with OXA-48 as the control group).

Two mutational targets identified in our study (F72L and L158P) were recently isolated from the environment, in combination with a second amino acid substitution (23). We aimed to elucidate the effect of G131S and N146S in combination with F72L and L158P, respectively. To do so, we competed F72L and L158P against the double mutants F72L/G131S and N146S/L158P, respectively. No significant change in fitness was detectable in the absence of selection pressure (Fig. 3b; Welch *t*-test, *P* = 0.24 and 0.62 for F72L/G131S and N146S/L158P, respectively). At sub-MIC ceftazidime, our data suggest no positive selection for the double mutants (Fig. 3b; paired Welch *t*-test between conditions, *P* = 0.038 and 0.59 for F72L/G131S and N146S/L158P, respectively). Thus, the role of these second mutations remains unclear.

### Mutations within *bla*_OXA-48_ alter enzymatic properties.

In this study, sub-MIC levels of ceftazidime were shown to select for OXA-48 variants conferring high bacterial fitness advantages despite only cryptic resistance phenotypes, suggesting that, in the clinical setting, these genetic changes are likely to remain undetected. To further our understanding of how these cryptic changes influence the enzymatic properties of OXA-48, we expressed the enzymes without their leader sequence in E. coli BL21 AI (Table S1). After enzyme purification, protein masses were verified using electrospray ionization mass spectrometry (ESI-MS). The molecular weight of five out of seven variants corresponded to their calculated monoisotopic masses (Table 3). For F156C and G160C, we observed an increase in molecular weight by 76 Da, likely caused by the β-mercaptoethanol used during the purification process to minimize aggregation.

**TABLE 3:** Overview of enzyme kinetic values, molecular weight, and thermal stability of OXA-48 and variants

OXA-48 variant	Calculated mol wt (Da)^*a*^	Measured mol wt (Da)^*a*^	Thermostability (°C)^*b*^	*k*_cat_/*K_m_* (mM^−1^ s^−1^) for:^*c*^					
				Ampicillin	Piperacillin	Ceftazidime	Cefepime^*f*^	Imipenem	Meropenem
Wild type	28,186.3	28,186.5	55.9	5.56 × 10^3^	2.26 × 10^3^	3.4 × 10^−1^	1.20 × 10^−1^	1.05 × 10^2^	4.58 × 10^1^
L67F	28,220.3	28,220.4	51.4	4.66 × 10^2^	7.80 × 10^0^	9.4 × 10^−1^	1.68 × 10^−2^	6.38 × 10^0^	7.81 × 10^−1^
P68S	28,176.3	28,176.3	48.3	1.80 × 10^2^	1.48 × 10^1^	1.6 × 10^0^	9.12 × 10^−2^	3.56 × 10^0^	3.20 × 10^0^
F72L	28,152.3	28,151.5	49.2	1.44 × 10^2^	1.35 × 10^1^	1.1 × 10^1^	8.81 × 10^−3^	3.38 × 10^0^	4.47 × 10^0^
F156C^*d*^	28,142.3	28,218.3	50.7	2.24 × 10^2^	9.39 × 10^0^	1.5 × 10^0^	2.42 × 10^−2^	8.17 × 10^−2^	6.54 × 10^−2^
F156V	28,138.3	28,138.5	50.2	1.18 × 10^2^	9.03 × 10^0^	1.5 × 10^0^	3.35 × 10^−2^	2.85 × 10^0^	6.10 × 10^−1^
L158P	28,170.3	28,170.5	50.3	2.37 × 10^2^	2.69 × 10^1^	6.7 × 10^−1^	ND	1.38 × 10^0^	4.56 × 10^−1^
G160C^*d*^	28,232.3	28,308.3	48.4	2.24 × 10^2^	1.40 × 10^1^	3.6 × 10^0^	1.27 × 10^−1^	4.36 × 10^0^	6.52 × 10^0^
F72L/G131S^*e*^	28,182.3	28,182.0	45.2	1.46 × 10^2^	3.07 × 10^1^	5.9 × 10^−1^	1.27 × 10^−1^	2.03 × 10^0^	8.94 × 10^0^
N146S/ L158P^*e*^	28,143.3	28,142.0	50.7	2.05 × 10^2^	2.87 × 10^1^	6.9 × 10^1^	ND	3.02 × 10^−1^	5.76 × 10^−1^

a Monoisotopic mass after TEV cleavage.

b Measured as thermostability.

c Michaelis-Menten kinetics could not be saturated for most of the variants; therefore, only *k*_cat_/*K_m_* values were reported.

d Purified in the presence of β-mercaptoethanol to minimize aggregation.

e Second mutations (G131S and N146S) described in environmental samples.

f ND, no activity detected.

We determined their catalytic efficiencies (*k*_cat_/*K_m_*) toward a panel of β-lactams and found that they were in line with the antimicrobial susceptibility data. Toward ceftazidime, *k*_cat_/*K_m_* values were increased by 2- to 31-fold (Table 3) for all variants compared to wild-type OXA-48. Moreover, all variants exhibited strongly reduced activity (up to several magnitudes) against penicillins (ampicillin and piperacillin) as well as toward carbapenems (meropenem and imipenem). To test for cross-activity against 4^th^-generation cephalosporins, we determined the catalytic efficiencies against cefepime. Also, here, we found that the OXA-48 variants tended to display *k*_cat_/*K_m_* values several magnitudes lower than the wild-type OXA-48 (Table 3).

Functional mutations within serine β-lactamases have frequently been described to decrease the thermostability (17, 25, 26). Indeed, compared to wild-type OXA-48, all single amino acid changes were deleterious with respect to thermostability, which decreased by 4.5 to 7.6°C (Table 3). F72L/G131S exhibited the lowest melting temperature, with a decrease of 10.7°C. Generally, we found the following order for the thermal stability OXA-48 > L67F > F156C = N146S/L158P > L158P = F156V > F72L > G160C = P68S > F72L/G131S.

### P68S, F72L, and L158P increase the loop flexibility within OXA-48.

Single amino acid changes in OXA-48 were responsible for increased catalytic activity against ceftazidime. To understand the underlying structural changes allowing these OXA-48 variants to hydrolyze ceftazidime more efficiently, we first mapped all amino acid changes onto the structure of OXA-48, showing that they clustered around the α3-helix (L67F, P68S, and F72L) and the Ω-loop (F156C, F156V, L158P, and G160C) (Fig. 2c). Second, multiple independent MD simulations were performed on a subset of variants (P68S, F72L, and L158P) with covalently bound ceftazidime in their active site. Our previous study showed that an amino acid change at position 68 (P68A) decreases ceftazidime susceptibility in OXA-48 (17). Additionally, positions 72 and 158 were selected due to amino acid changes recently identified in environmental samples (F72I and L158P). Changes in enzyme flexibility were analyzed by calculating root mean square fluctuations (RMSF) for the backbone atoms in the Ω- and β5-β6 loops and compared to wild-type OXA-48 and the ceftazidimase OXA-163 (only Ω-loop, due to the shortened β5-β6 loop).

**FIG 4:**
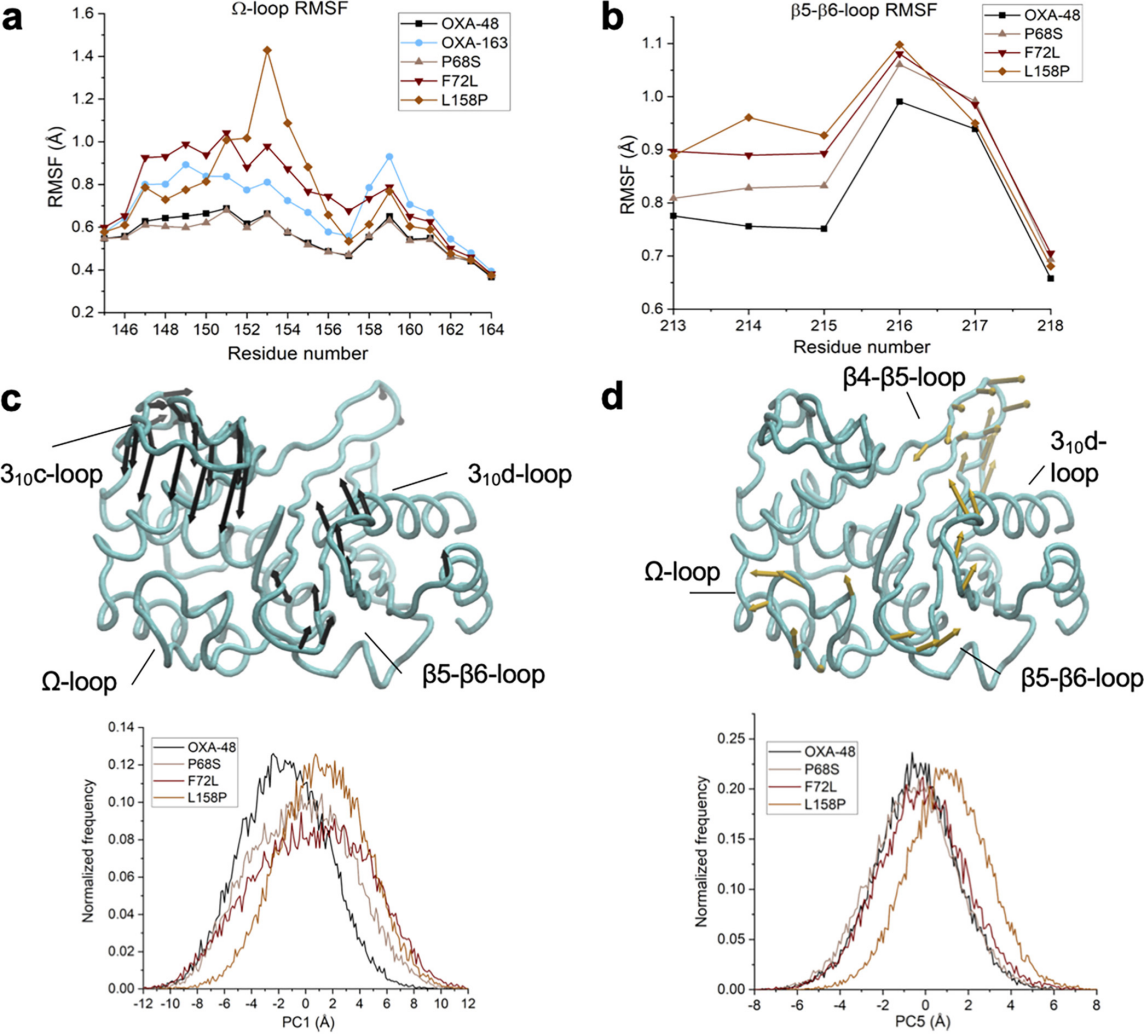
Differences in dynamics between wild-type OXA-48 and variants P68S, F72L, and L158P (as well as OXA-163). Loop flexibility as measured by backbone root mean square fluctuations (RMSFs) from molecular dynamics simulations for residues in the Ω-loop (a) and in the b5-b6 loop (b). Principal component (PC) analysis of the Cα atoms from molecular dynamics simulations of OXA-48 and the P68S, F72L, and L158P variants, with PC1 (c, top) and PC5 (d, top) illustrated on ribbon structures using arrows indicating the direction of the eigenvectors and the magnitude of the corresponding eigenvalue (for clarity, arrows are only shown for atoms with eigenvalues of >2.5 Å). Normalized histograms (using 200 bins per enzyme) for PC1 (c, bottom) and PC5 (d, bottom) indicate differences of the range of the PC sampled in different variants.

For the Ω-loop, P68S displayed very similar RMSF values relative to OXA-48; however, F72L and L158P showed increased flexibility in this region, displaying even higher RMSF values than OXA-163 (Fig. 4a). Notably, the L158P substitution increased fluctuations specifically for residues N152 to S155. V153 demonstrated the largest overall shift in RMSF values, with an increase of 0.7 Å compared to OXA-48. For the β5-β6 loop, all variants exhibited an increase in fluctuations, especially for the residues T213 to E216 (Fig. 4b).

Possible changes in intramolecular interactions due to the amino acid changes P68S, F72L, and L158P were also studied from MD simulations. For P68S, an H-bond was observed between the hydroxyl groups of S68 and Y144 (Fig. 2d). However, no other apparent structural changes near the active site were directly observed, and therefore, the effect of P68S on the dynamic nature remains elusive.

In wild-type OXA-48, W157 in the Ω-loop stacks with both F72 and F156 (Fig. 2d). Consequently, the lack of this interaction in F72L likely increases the flexibility of W157, which is reflected by a 0.2-Å increase in calculated RMSF (Fig. 4a). Furthermore, the wild-type Ω-loop displays an organized H-bond network, which extends to R128 and R214 on either side (Fig. 2d). We found that L158P is likely affecting this network by disrupting the interactions with S155 and D159 (Fig. 2d). Consequently, the salt bridge between R128 and D154 was found to be weakened, as its presence was reduced from 87% to 43% of the simulation time. The loss of the backbone H-bond between L158 and S155, in the proline variant (L158P), has a knock-on effect on the rest of the loop, making it more flexible and likely to better accommodate bulkier β-lactam substrates such as ceftazidime.

Aside from flexibility and changes in amino acid interactions, possible further effects on the overall enzyme dynamics were inspected by performing principal component (PC) analysis on the combined MD trajectories (using the Cα-atom positions). The overall sampling of conformational space is highly similar for OXA-48 and the three variants. There are no specific large conformational changes or coordinated loop movements induced by the mutations. Some differences between variants and wild-type OXA-48 were observed particularly for PCs that primarily involve movement of loops, including those surrounding the active site, further indicating that the mutations introduce small changes in loop dynamics (Fig. 4c and d).

### Cryptic binding pocket for ceftazidime in OXA-48.

**FIG 5:**
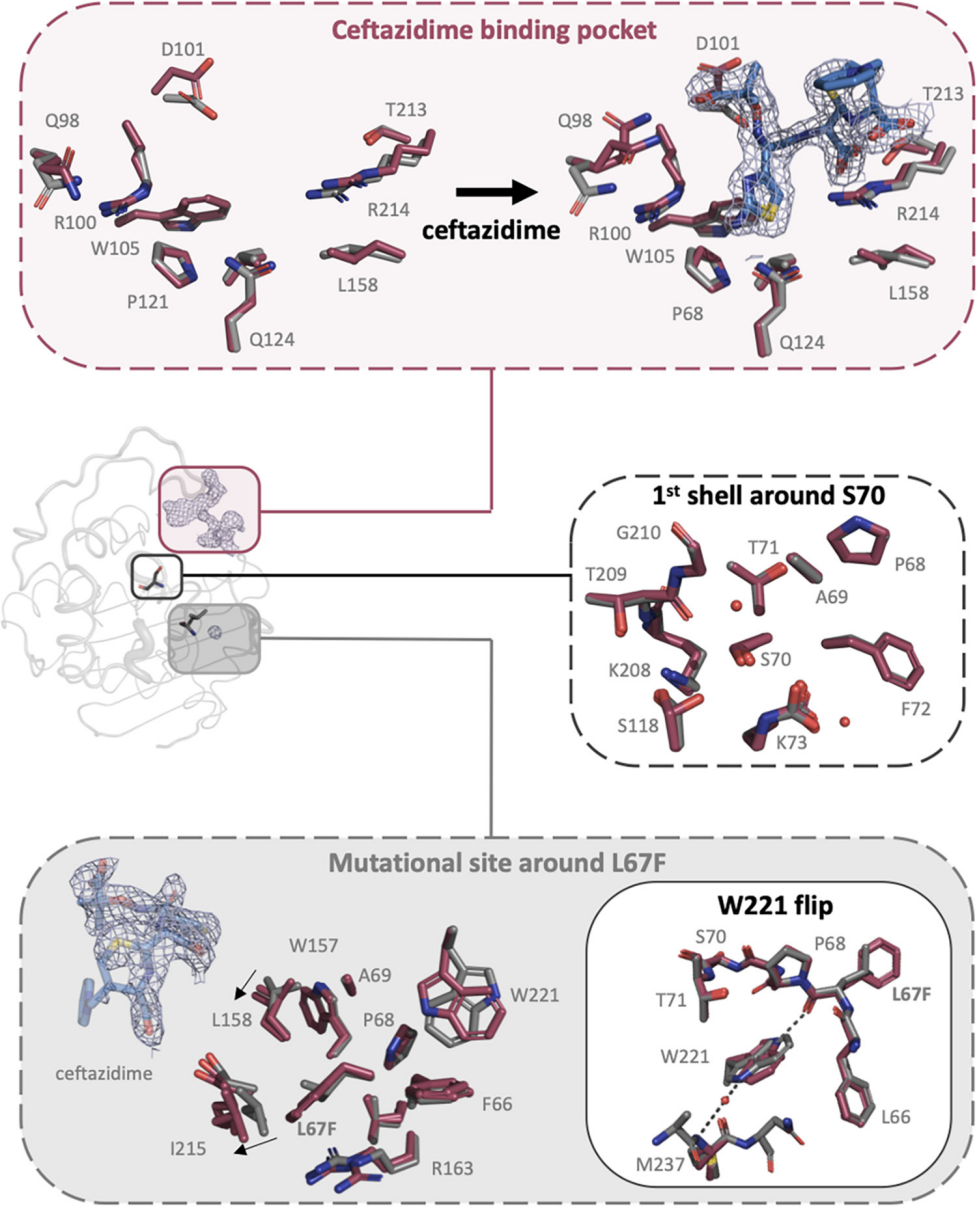
Ceftazidime binding pocket (top panel), the 1st shell residues interacting with S70 (middle) and the mutational site (bottom panel) of L67F (red) compared to the wild-type structure of OXA-48, shown in gray (PDB no. 3HBR) (15). The crystal structure of L67F was solved to 1.9 Å and displayed hydrolyzed ceftazidime ∼9 Å away from the active site S70. (Top) Binding pocket of L67F without (left, chain C) and with (right, chain A) ceftazidime compared to wild-type OXA-48. For ceftazidime, no 2Fo-Fc electron density was detected for the R2 group. (Middle) Superimposition of the first shell residues of L67F (chain A) around the active site S70 compared the wild-type structure. (Bottom) Investigation of the mutational site, shown as the first shell residues around L67F (both chain A and C), compared to wild-type OXA-48. Displacements of L158 (1 Å) and I215 (2 Å) in the L67F structure are indicated with arrows. W221 was flipped 180° in the L67F structure.

To investigate substrate binding, all OXA-48 variants were crystalized and soaked with ceftazidime. We were able to solve the crystal structure of L67F to 1.9 Å with four chains (A to D) in the asymmetric unit (space group P2_1_2_1_2_1_), which were arranged into two dimers (chains A/C and B/D). Chain C and D carried a hydrolyzed ceftazidime molecule approximately 9 Å away from the active site S70. The R1-group of ceftazidime including the dihydrothiazine ring demonstrated clear electron density (2Fo-Fc); however, no electron density was observed for the R2-ring (Fig. 5, top).

**FIG 6:**
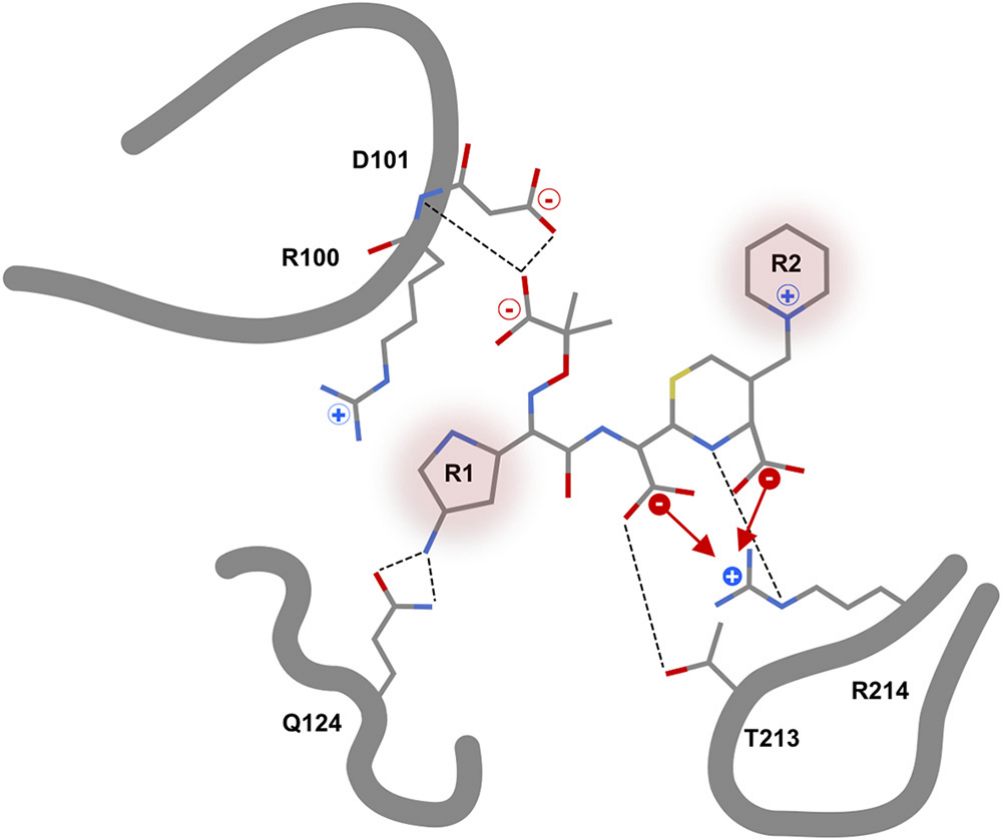
Schematic representation of hydrolyzed ceftazidime in front of the active site of the OXA-48 variant L67F (based on PDB no. 7ASS). The ceftazidime side chains R1 and R2 are labeled and marked. For R2, no electron density was observed and therefore no interactions were detected. Hydrogen bonds from ceftazidime to D101, Q124, T213, and R214 are represented with dashed lines. The ionic interactions with R214 are indicated with arrows.

We first investigated the binding of ceftazidime to the L67F variant. Here, we found that Q98, R100, D101, W105, V120, P121, Q124, L158, T213, and R214 formed a cryptic pocket outside the active side and that these residues were involved in stabilizing the hydrolyzed ceftazidime molecule (Fig. 5, top). D101, Q124, T213, and R214 were found to interact with ceftazidime via H-bonds. R214 was further involved in ionic interactions with two of the carboxylic acid groups of hydrolyzed ceftazidime (Fig. 6).

Second, we investigated the active site architecture, including the first shell residues around S70. Chain A was therefore superimposed onto a wild-type structure of OXA-48 (PDB no. 3HBR) (15). As expected, superimposition resulted in low root mean square deviations of 0.21 Å. We found that K73 was carboxylated and that all first shell residues nicely aligned with the wild-type structure (Fig. 5, middle).

Third, we investigated the mutational site around L67F that is located “below” the active site (Fig. 5, bottom). We found F66, P68, A69, W157, L158, R163, I215, and W221 to be directly interacting with amino acid position 67. In the L67F variant, both with and without ceftazidime, L158 and I215 were shifted by 1 to 2 Å, respectively. In addition, we found W221 to be flipped by 180°. While in the wild-type structure, the W221 side chain forms a water-mediated H-bond to the backbone nitrogen of M237, in L67F, W221 formed a H-bond to the main chain of F67 (Fig. 5).

## DISCUSSION

Here, we asked if sub-MICs of the clinically relevant β-lactam ceftazidime could affect the evolution of the contemporary, globally circulating carbapenemase OXA-48. To test this, we evolved an OXA-48 producing E. coli strain in the presence of one-quarter of its ceftazidime MIC. The identification of seven single variants of OXA-48, conferring only marginal changes in susceptibility (Fig. 2b, Table 2), demonstrates that the exposure to sub-MICs of ceftazidime drives the emergence of cryptic *bla*_OXA-48_ genetic diversity. Thus, our data provide further support for the proposed compartment hypothesis (6, 8, 27, 28), where low-grade selection promotes cryptic genetic variation that could act as stepping-stones toward full clinical antibiotic resistance (29, 30). Notably, even though all seven single OXA-48 variants largely displayed, from a clinical microbiology perspective, negligible changes in ceftazidime susceptibility, competition experiments revealed strong beneficial fitness effects (Fig. 2b). Taken together with earlier work, using reconstructed TEM-1 variants from clinical samples (5), our data underscore the significance of divergent evolution and selection of genetic variation imposed by sub-MICs of β-lactams.

To further our understanding of how the detected single amino changes affect the structure-activity relationship of OXA-48, we first measured enzyme kinetics (Table 3). The catalytic efficiency mirrored the observed changes in susceptibility toward β-lactams at the cellular level and confirmed our previous findings that mutational changes increasing ceftazidime activity come with a functional trade-off against penicillins and carbapenems (17). However, decreased susceptibility might not only be reflected by differences in the catalytic efficiency, as protein stability and translocation also affect resistance levels toward β-lactams (31). Structurally, all amino acid changes clustered either around the active site S70 (L67F, P68S, F72L) or within the Ω-loop (F156C, F156V, L158P, G160C; Fig. 2c).

In wild-type OXA-48, the Ω-loop interacts with the β5-β6 loop via a salt bridge mediated by D159-R214 maintaining a closed conformation of the active site (15). MD simulations performed on a subset of variants revealed that F72L and L158P weaken the interaction between these loops, resulting in increased structural flexibility (Fig. 4). We postulate that these changes aid the hydrolysis of bulkier substrates such as ceftazidime but result in decreased activity toward penicillins and carbapenems. Indeed, mutations affecting the salt bridge are associated with reduced carbapenemase activity presumably due to increased loop flexibility (32).

In clinical OXA-48-like variants (e.g., OXA-163, OXA-247, and OXA-405) with increased ceftazidime activity, larger structural variations (deletions in combination with single point mutations) within and around the β5-β6 loop have been reported (18–20). However, also, single amino acid changes structurally close to the Ω- and β5-β6 loops have been shown to slightly elevate the catalytic efficiency toward ceftazidime (E125Y in OXA-245 and V120L in OXA-519) (33, 34). The significance of the Ω- and β5-β6 loops for the substrate profile is not limited to OXA-48 (35, 36). These loops have been shown to impact substrate profiles for other clinically relevant β-lactamases, including TEM and KPC (37–39). In addition, comparable modes of action have been hypothesized for the OXA-10-like variants OXA-145 and OXA-147, exhibiting L158Δ and W157L, respectively (according to OXA-48 numbering) (40–42).

We were able to solve the structure of the OXA-48 variant L67F, which showed the reorientation of several active site residues such as L158 and I215 (Fig. 5, bottom). Fine-tuning of active site residue conformations through more distal amino acid changes is a common strategy in enzyme evolution (43) and may enable L67F to increase activity toward ceftazidime. In addition, our structure revealed the presence of a new cryptic binding site for ceftazidime, approximately 9 Å away from the active site residue S70, involving interaction with the above-described β5-β6 loop (Fig. 5, bottom) and R214, in particular. However, the mechanistic importance of this binding and the pocket remains elusive, and more structural investigations are necessary.

Taken together, combining experimental evolution and structure-activity relationships allowed us to identify and characterize single step mutations with cryptic resistance that yet demonstrated significant fitness effects and structural changes. Our data show that to understand the evolutionary potential of standing genetic diversity, susceptibilities characterized solely by traditional MIC measurements provide too low resolution.

We acknowledge that our study is not without limitations, as despite strong fitness effects, none of the variants went to fixation in any of the populations (Fig. 1c). We argue that there can be at least two reasons for this. First, we have focused solely on changes within OXA-48 and on a single drug. It is clear from our data that the evolution at sub-MICs also selected for other potential resistance mechanisms (e.g., efflux, porins). These mechanisms could lead to potential widespread epistatic interactions that would slow down any fixation process (9, 11, 44–46) and warrant further investigations, e.g., whole-genome sequencing. Second, it has been shown that β-lactamase producers can detoxify their environment, allowing coexistence of genotypes with different susceptibilities, resulting in a stable equilibrium between resistant and susceptible clones (47).

Our work sheds light on the evolution of β-lactamases and their selection dynamics toward altered substrate profiles. This is supported by recent studies reporting environmental contamination of cephalosporins at concentrations similar to those applied here (48, 49). Moreover, OXA-48 variants, with the same or similar amino acid changes as identified and characterized here, have been reported in environmental samples (22, 50). We speculate that the identified mutations are only first step mutations toward full clinical ceftazidime resistance mediated by OXA-48. However, more studies are needed to fully understand the complete fitness landscape of OXA-48 and other carbapenemases.

## MATERIALS AND METHODS

### Media, chemicals, and strains.

Mueller-Hinton (MH) agar and broth were purchased from Thermo Fisher Scientific (East Grinstead, UK). Luria-Bertani (LB) broth, LB agar, yeast extract, agar, terrific broth, ampicillin, amoxicillin, cefepime, ceftazidime, chloramphenicol, imipenem, meropenem, piperacillin, 2,3,5 tri-phenyl tetrazolium, sodium chloride, and maltose were obtained from Sigma-Aldrich (St. Louis, MO, USA). Tryptone was obtained from Oxoid (Hampshire, UK). All strains used and constructed within this study are listed in Table S1.

### Sub-MIC evolution.

MP13-06, previously constructed and tested (17), was evolved by serial passaging without selection pressure and at 0.25× MIC (0.06 mg/liter) of ceftazidime for 300 generations. In short, bacterial suspensions were grown at 37°C and 700 rpm on a plate shaker (Edmund Bühler, Bodelshausen, Germany) in 1 ml MH broth to full density and passaged every 12 h with a bottleneck of 1:100. The evolution was performed in triplicates.

### Dose-response curves and susceptibility testing.

Dose-response curves were obtained initially and after every 50 generations for the whole evolved populations. Cultures were grown to full density and diluted in 0.9% saline to 10^6^ CFU/ml. Then, 384-well plates (VWR, Radnor, PA, USA) were inoculated with 10^5^ CFU and increasing concentrations of ceftazidime ranging from 0 to 32 mg/liter. Plates were statically incubated for 20 h at 37°C. The optical density at 600 nm (OD_600_) was measured with a microtiter plate reader (BioTek Instruments, Winooski, VT, USA), and dose-response curves including IC_50_ values were calculated using Prism 9.0 (GraphPad Software, San Diego, CA, USA). In this setup, the ceftazidime MIC was determined by measuring the OD_600_ as the first well with an optical density comparable to the negative control.

For MIC measurements against β-lactams other than ceftazidime, in-house-designed and premade Sensititre microtiter plates (TREK Diagnostic Systems/Thermo Fisher Scientific, East Grinstead, UK) were loaded with 10^5^ CFU. The plates were incubated statically for 20 h at 37°C. All susceptibility tests were performed in at least two biological replicates.

### Determination of clones with altered ceftazidime susceptibility.

To determine clones exhibiting decreased ceftazidime susceptibility, we plated 10^7^ cells from every 50th generation on MH agar without and with 1 mg/liter ceftazidime. The plates were incubated for 24 h at 37°C. Clone frequencies were determined as the ratio between colonies found on selective versus nonselective plates. About 50 preselected colonies were subjected to susceptibility testing, as described above. For creating the boxplots, a probability function was calculated based on the MIC per replicate. Since the MICs were determined in 2-fold steps, we generated smoother boxplots by creating 1,000 random measurements per generation. We did so by drawing a random number between the log_2_ (MIC values) and log_2_ (MIC values) + 1 a 1,000 × f_mic, where f_mic is the fraction of the population. All calculations were done in Mathematica 11.0 (Wolfram Research, Champaign, IL, USA).

### Strain construction.

For functional resistance profiles, wild-type *bla*_OXA-48_ and allele variants were subcloned into the high copy number vector pCR-blunt II-TOPO vector (Invitrogen, Carlsbad, CA, USA) and expressed in E. coli TOP10 (Invitrogen). For wild-type TOPO-*bla*_OXA-48_, the construction has been described previously (17). Point mutations were inserted by using the QuikChange II kit for site-directed mutagenesis (Agilent Biosciences, Santa Clara, CA, USA), TOPO-*bla*_OXA-48_ as a template, and the respective primers (Table S2). The double mutants TOPO-*bla*_OXA-48_-F72L/G131S and TOPO-*bla*_OXA-48_-N146S/L158P were created by inverse PCR using Phusion polymerase (New England Biolabs, Ipswich, MA, USA) and TOPO-*bla*_OXA-48_-F72L or TOPO-*bla*_OXA-48_-L158P as a template, respectively. PCR products were 5′-phosphorylated with polynucleotide kinase (Thermo Fisher Scientific, Waltham, MA, USA) and circularized using T4 DNA ligase (Thermo Fisher Scientific). Transformants were selected on LB agar plates containing 50 or 100 mg/liter ampicillin. *Bla*_OXA-48_ was Sanger sequenced (BigDye 3.1 technology; Applied Biosystems, Foster City, CA, USA) using M13 primers (Thermo Fisher Scientific) (Table S2).

10.1128/mSphere.00108-21.2 TABLE S2 Primers used in the study Table S2, DOCX file, 0.02 MB. Copyright © 2021 Fröhlich et al. 2021 Fröhlich et al. https://creativecommons.org/licenses/by/4.0/ This content is distributed under the terms of the Creative Commons Attribution 4.0 International license.

For expression in a low copy number vector (pUN), we PCR-amplified a segment containing the p15A origin of replication and the *cat* chloramphenicol resistance gene of the pACYC184 vector using the primers cat-r and p15A46 (Table S2). To obtain the *bla*_OXA-48_ inserts, the pCR-blunt II-TOPO constructs (see above) were used as templates. We amplified the *bla*_OXA-48_ genes by using the primers OXA-48-pro-f, containing the constitutive artificial CP6 promoter (51), and preOXA-48B (Table S2) (52). These PCR products were 5′-phosphorylated with polynucleotide kinase (Thermo Fisher Scientific) and then blunt ligated with the amplified vector backbone. The pUN-*bla*_OXA-48_ vector and the corresponding variants were transformed into E. coli DH5α and plated on LB agar containing chloramphenicol (25 mg/liter). Genotypes of selected clones were confirmed by Sanger sequencing (BigDye 3.1 technology; Applied Biosystems) using OXA-48-pro-f/preOXA-48B primers (Table S2) (52).

To measure bacterial fitness, E. coli MG1655 *ΔmalF* (MP14-23) was constructed as a competitor strain by transducing the kanamycin resistance marker from the Keio strain JW3993 with P1-vir into E. coli MG1655 as previously described (53, 54). The marker was then removed with the helper vector pCP20 (55). The competitor strain MP14-23 was then transformed with pUN-*bla*_OXA-48_ and the corresponding variants (Table S1). Transformants were selected on LB plates containing 25 mg/liter chloramphenicol.

For protein expression and purification, *bla*_OXA-48_ in the pDEST17 expression vector (Thermo Fisher Scientific) was mutagenized using a QuikChange II site-directed mutagenesis kit as described above. E. coli DH5α was transformed with the DNA constructs, and clones were selected on LB agar containing 100 mg/liter ampicillin. The vectors were isolated using a plasmid maxi kit (Qiagen, Hilden, Germany) and transformed into E. coli BL21 AI (Thermo Fisher Scientific). Point mutations were verified by Sanger sequencing using T7 primers (Thermo Fisher Scientific) (Table S2).

### Bacterial fitness: head-to-head competition.

Strains were grown overnight in LB supplemented with chloramphenicol (25 mg/liter) at 37°C and 700 rpm on a plate shaker (Edmund Bühler). For each competition, we coinoculated ∼1 × 10^7^ CFU/ml of each competitor in 1 ml LB broth, supplemented either with chloramphenicol (25 mg/liter) and ceftazidime (0.06 mg/liter) or with chloramphenicol only. Then, 96-deep-well plates (VWR) were incubated at 37°C and 700 rpm for 8 h. Each competition was performed in three biological replicates. The initial and final CFU/ml for both competitors were determined by differential plating on tetrazolium maltose agar (10 g/liter tryptone, 5 g/liter sodium chloride, 1 g/liter yeast extract, 15 g/liter agar, 10 g/liter maltose, supplemented with 1 ml 5% 2,3,5 tri-phenyl tetrazolium chloride). Relative fitness (w) was determined according to equation 1, where *mal^+^* and *mal*^−^ are, respectively, the *mal^+^* and Δ*malF* strain backgrounds carrying the different pUN vectors (Table S1).

(1) $$w=\frac{\log_{2}\frac{{\text{mal}}_{\text{final}}^{-}}{{\text{mal}}_{\text{initial}}^{-}}}{\log_{2}\frac{{\text{mal}}_{\text{final}}^{+}}{{\text{mal}}_{\text{initial}}^{+}}}$$

The *mal^+^* pUN-*bla*_OXA-48_ strain (MP08-61) was competed against Δ*malF* strains carrying each of the pUN vectors encoding wild-type *bla*_OXA-48_ or single variants (Table S1). Additionally, the *mal^+^* pUN-*bla*_OXA-48_-F72L (MP08-67) and *mal^+^* pUN-*bla*_OXA-48_-L158P (MP08-63) strains were used as competitors, respectively, against strains Δ*malF* pUN-*bla*_OXA-48_-F72L/G131S (MP14-32) and Δ*malF* pUN-*bla*_OXA-48_-N146S/L158P (MP14-33), respectively. Data analysis and graphical illustrations were performed in R 4.0.2 (56).

### Recombinant enzyme expression and purification.

Overexpression of OXA-48 and the corresponding variants was done in terrific broth supplemented with 100 mg/liter ampicillin. E. coli BL21 AI carrying pDEST-17-*bla*_OXA-48_ and OXA-48 variants (Table S1) were grown at 37°C and 220 rpm to an optical density of 0.4 to 0.5. Protein expression was induced with 0.1% l-arabinose (Sigma-Aldrich). Expression took place for 16 h at 15°C and 220 rpm. Harvested cells were sonicated, and recombinant proteins were purified as described previously (17, 57). F156C and G160C were found to be insoluble. To increase their solubility, 5 mM β-mercaptoethanol was used during the sonication process.

### Molecular mass verification.

ESI-MS was performed on the purified enzymes as described previously (58). In short, a buffer exchange to 0.1% formic acid (Merck Millipore, Burlington, MA, USA) was performed using centrifugal molecular cutoff filters (Merck Millipore; 10,000 Da). The protein masses were determined using an Orbitrap Fusion Lumos device (Thermo Fisher Scientific). Injection was performed using an EASY-nano LC instrument (Thermo Fisher Scientific) with a 15-cm C_18_ EASY-spray column. Mass calculations were done using BioPharma Finder 3.0 protein deconvolution software (Thermo Fisher Scientific, Massachusetts, USA).

### Steady-state enzyme kinetics.

Catalytic efficiencies (*k*_cat_/*K_m_*) for the recombinantly expressed enzymes were determined under steady-state conditions for ampicillin (Δξ = −820 M^−1^ cm^−1^, 232 nm), piperacillin (Δξ = −820 M^−1^ cm^−1^, 235 nm), ceftazidime (Δξ = −9,000 M^−1^ cm^−1^, 260 nm), cefepime (Δξ = −10,000 M^−1^ cm^−1^, 260 nm), imipenem (Δξ = −9,000 M^−1^ cm^−1^, 300 nm), and meropenem (Δξ = −6,500 M^−1^ cm^−1^, 300 nm) by measuring the initial enzymatic reaction rate. The enzyme concentrations are summarized in Table S3. All determinations were performed at least in duplicates at a final assay volume of 100 μl. UV-transparent 96-well plates (Corning, Kennebunk, ME, USA) were used. All test results were obtained at 25°C and in 0.1 M phosphate buffer (pH 7.0) supplemented with 50 mM NaHCO_3_ (Sigma-Aldrich). Calculations were performed using Prism 9.0 (GraphPad Software).

10.1128/mSphere.00108-21.3 TABLE S3 Enzyme concentrations (nM) for steady-state kinetics Table S3, DOCX file, 0.01 MB. Copyright © 2021 Fröhlich et al. 2021 Fröhlich et al. https://creativecommons.org/licenses/by/4.0/ This content is distributed under the terms of the Creative Commons Attribution 4.0 International license.

### Thermostability.

We determined the fluorescence-based protein thermostability for OXA-48 as described previously (17). In short, the proteins were diluted in 50 mM HEPES (VWR), pH 7.5 supplemented with 50 mM potassium sulfate (Honeywell, North Carolina, USA) to a final concentration of 0.2 mg/ml protein and 5× SYPRO orange (Sigma-Aldrich). A temperature gradient of 25 to 70°C (heating rate, 1°C per min) was applied using an MJ minicycler (Bio-Rad, Hercules, CA, USA). All experiments were performed in triplicates.

### Molecular dynamics simulations.

System setup was performed as described previously (59). In brief, acyl-enzyme of OXA-48 with covalently bound ceftazidime was originally built using the structure of OXA-48 with imipenem (PDB no. 5QB4) (60) and replacing imipenem with ceftazidime (PDB no. 6Q5F) (17), which ensured keeping the Ω-loop ordered (as found in the apoenzyme). For OXA-163, the same ceftazidime binding pose was combined with apoenzyme crystal structure (PDB no. 4S2L) (61). All OXA-48 variants were modeled using the mutagenesis tool in PyMOL, choosing the rotamer with the least steric clashes with surrounding atoms. Enzymes were solvated in a 10-Å cubic box of TIP3P water with net charge neutralized by replacing bulk water molecules with counterions. Parameters for nonstandard residues (carboxylated K73, ceftazidime acyl-enzyme) have been published previously (59).

All systems were initially briefly minimized (1,000 steps of steepest descent followed by 1,000 steps of conjugate gradient), heated from 50 K to 300 K in 20 ps, and then simulated for 120 ns in the NPT ensemble (saving a frame every 20 ps). Langevin dynamics were used with a collision frequency of 0.2 and a 2-fs time step; all bonds involving hydrogens were constrained using the SHAKE algorithm. Periodic boundary conditions in explicit solvent were applied in all simulations. Five independent simulations were run per enzyme variant (for a total of 600 ns per variant), and all calculations were done with the Amber18 program package (pmemd.cuda) (62) using the ff14SB force field (63) for the protein and TIP3P for water (64, 65). All analyses were done using CPPTRAJ from AmberTools (66). Root mean square fluctuation (RMSF) calculations were performed by first calculating an average structure from the MD simulation, aligning the trajectory against the average structure excluding four residues from both the N- and C-terminal ends as well as the loop residues of interest (G145-I164 for the Ω-loop and T213-I219 for the β5-β6-loop), and then calculating the RMSF for the corresponding loop main chain heavy atoms (CA, C, N, O). The first 20 ns were excluded from all analyses to allow time for system equilibration. PC analysis was performed on Cα-atoms (excluding four residues from both the N- and C-terminal ends), using all trajectories of OXA-48 and variants together (5 independent simulations of 120 ns per variant).

### Crystallization and structure determination.

Crystals were grown in a 1-μl hanging drop containing 5 mg/ml enzyme and mixed 1:1 with reservoir solution containing 0.1 M Tris, pH 9.0 (Sigma-Aldrich), and 28 to 30% polyethylene glycol (PEG) mono ethylene ether 500 (Sigma-Aldrich) at 4°C. Crystals were harvested, cryoprotected by adding 15% ethylene glycol (Sigma-Aldrich) to the reservoir solution, and then frozen in liquid nitrogen.

Diffraction data were collected on BL14.1 BESSY II, Berlin, Germany, at 100 K, wavelength 0.9184 Å, and the diffraction images were indexed and integrated using XDS (67). AIMLESS was used for scaling (68). When scaling the final data set (Table S4), we aimed for high overall completeness and CC_1/2_ > 0.5 and a mean intensity above 1.0 in the outer resolution shell. The structure was solved by molecular replacement with chain A of PDB no. 5QB4 (60) and the program PHENIX 1.12 (69). Parts of the model were rebuilt using Coot (70). Figures were prepared using PyMOL 1.8 (Schrödinger, New York, NY, USA). Ligand and protein interactions were calculated using Protein Contacts Atlas (71).

10.1128/mSphere.00108-21.4 TABLE S4 X-ray data collection and refinement statistics for the OXA-48 variant L67F in complex with hydrolyzed ceftazidime (values in parenthesis are for the highest-resolution shell) Table S4, DOCX file, 0.02 MB. Copyright © 2021 Fröhlich et al. 2021 Fröhlich et al. https://creativecommons.org/licenses/by/4.0/ This content is distributed under the terms of the Creative Commons Attribution 4.0 International license.

### Data availability.

Atom coordinates and structure factors for the OXA-48 variant L67F are available in the protein data bank (PDB no. 7ASS). Underlying data are stored in an online repository at https://github.com/GAMMAtri/Cryptic_b-lactamase_evolution.
