# Supplementary material for: Cryptic β-Lactamase Evolution Is Driven by Low β-Lactam Concentrations
Source: mSphere. 2021 Apr 28;6(2):e00108-21. doi: 10.1128/mSphere.00108-21 (PMC8092134; doi:10.1128/mSphere.00108-21)
Supplement: TABLE S1 [file mSphere.00108-21-st001.docx]

| *E. coli* Strain(s) | Comment | Source/  Reference |
| --- | --- | --- |
| MP13-01/  MP08-01 | K12 MG1655 | Uppsala/Lisbon University |
| MP13-06^a^ | MP13-01 transformed with p50579417_3_OXA-48 | (1) |
| MP13-34 | MP13-06 evolved in MH broth, population 1: 50 generations | this study |
| MP13-35 | MP13-06 evolved in MH broth, population 1: 100 generations | this study |
| MP13-36 | MP13-06 evolved in MH broth, population 1: 150 generations | this study |
| MP13-37 | MP13-06 evolved in MH broth, population 1: 200 generations | this study |
| MP13-38 | MP13-06 evolved in MH broth, population 1: 250 generations | this study |
| MP13-39 | MP13-06 evolved in MH broth, population 1: 300 generations | this study |
| MP13-40 | MP13-06 evolved in MH broth, population 2: 50 generations | this study |
| MP13-41 | MP13-06 evolved in MH broth, population 2:100 generations | this study |
| MP13-42 | MP13-06 evolved in MH broth, population 2:150 generations | this study |
| MP13-43 | MP13-06 evolved in MH broth, population 2: 200 generations | this study |
| MP13-44 | MP13-06 evolved in MH broth, population 2: 250 generations | this study |
| MP13-45 | MP13-06 evolved in MH broth, population 2: 300 generations | this study |
| MP13-46 | MP13-06 evolved in MH broth, population 3: 50 generations | this study |
| MP13-47 | MP13-06 evolved in MH broth, population 3: 100 generations | this study |
| MP13-48 | MP13-06 evolved in MH broth, population 3: 150 generations | this study |
| MP13-49 | MP13-06 evolved in MH broth, population 3: 200 generations | this study |
| MP13-50 | MP13-06 evolved in MH broth, population 3: 250 generations | this study |
| MP13-51 | MP13-06 evolved in MH broth, population 3: 300 generations | this study |
| MP13-52 | MP13-06 evolved in ceftazidime, population 4: 50 generations | this study |
| MP13-53 | MP13-06 evolved in ceftazidime, population 4: 100 generations | this study |
| MP13-54 | MP13-06 evolved in ceftazidime, population 4: 150 generations | this study |
| MP13-55 | MP13-06 evolved in ceftazidime, population 4: 200 generations | this study |
| MP13-56 | MP13-06 evolved in ceftazidime, population 4: 250 generations | this study |
| MP13-57 | MP13-06 evolved in ceftazidime, population 4: 300 generations | this study |
| MP13-58 | MP13-06 evolved in ceftazidime, population 5: 50 generations | this study |
| MP13-59 | MP13-06 evolved in ceftazidime, population 5: 100 generations | this study |
| MP13-60 | MP13-06 evolved in ceftazidime, population 5: 150 generations | this study |
| MP13-61 | MP13-06 evolved in ceftazidime, population 5: 200 generations | this study |
| MP13-62 | MP13-06 evolved in ceftazidime, population 5: 250 generations | this study |
| MP13-63 | MP13-06 evolved in ceftazidime, population 5: 300 generations | this study |
| MP13-64 | MP13-06 evolved in ceftazidime, population 6: 50 generations | this study |
| MP13-65 | MP13-06 evolved in ceftazidime, population 6: 100 generations | this study |
| MP13-66 | MP13-06 evolved in ceftazidime, population 6: 150 generations | this study |
| MP13-67 | MP13-06 evolved in ceftazidime, population 6: 200 generations | this study |
| MP13-68 | MP13-06 evolved in ceftazidime, population 6: 250 generations | this study |
| MP13-69 | MP13-06 evolved in ceftazidime, population 6: 300 generations | this study |
| For MIC measurements (high copy number vector): | | |
| MP13-04 | TOP10, recipient strain for pCR-blunt II-TOPO | Invitrogen |
| MP13-11 | MP13-04, transformed with pCR-blunt II-*bla*_OXA-48_ | (1) |
| MP13-21 | MP13-04, transformed with pCR-blunt II-*bla*_OXA -48_-L67F | this study |
| MP13-16 | MP13-04, transformed with pCR-blunt II-*bla*_OXA -48_-P68S | this study |
| MP13-14 | MP13-04, transformed with pCR-blunt II-*bla*_OXA-48_-F72L | this study |
| MP13-17 | MP13-04, transformed with pCR-blunt II-*bla*_OXA -48_-F156C | this study |
| MP13-18 | MP13-04, transformed with pCR-blunt II-*bla*_OXA -48_-F156V | this study |
| MP13-15 | MP13-04, transformed with pCR-blunt II-*bla*_OXA-48_-L158P | this study |
| MP13-19 | MP13-04, transformed with pCR-blunt II-*bla*_OXA-48_-G160C | this study |
| MP13-33 | MP13-04, transformed with pCR-blunt II-*bla*_OXA -48_-F72L/G131S | this study |
| MP13-20 | MP13-04, transformed with pCR-blunt II-*bla*_OXA -48_-N146S/L158P | this study |
| For dose-response measurements and head-to-head competitions (low copy number vector): | | |
| JW3393 | K12 BW25113, *ΔmalF*729::*kan* | (2) |
| MP14-23 | MG1655 *∆malF* constructed based on MP08-01 (*mal^+^*) | this study |
| MP08-61 | MP08-01 with pUN-*bla*_OXA-48_ | this study |
| MP14-24 | MP14-23 with pUN-*bla*_OXA -48_ | this study |
| MP14-29 | MP14-23 with pUN-*bla*_OXA -48_-L67F | this study |
| MP14-26 | MP14-23 with pUN-*bla*_OXA -48_-P68S | this study |
| MP14-27 | MP14-23 with pUN-*bla*_OXA -48_-F72L | this study |
| MP14-30 | MP14-23 with pUN-*bla*_OXA -48_-F156C | this study |
| MP14-31 | MP14-23 with pUN-*bla*_OXA -48_-F156V | this study |
| MP14-25 | MP14-23 with pUN-*bla*_OXA -48_-L158P | this study |
| MP14-28 | MP14-23 with pUN-*bla*_OXA -48_-G160C | this study |
| MP14-32 | MP14-23 with pUN-*bla*_OXA -48_-F72L/G131S | this study |
| MP08-67 | MP08-01 with pUN-*bla*_OXA -48_-F72L | this study |
| MP14-33 | MP14-23 with pUN-*bla*_OXA -48_-N146S/L158P | this study |
| MP08-63 | MP08-01 with pUN-*bla*_OXA -48_-L158P | this study |
| For protein expression and purification: | | |
| MP13-02 | BL21 AI recipient for pDEST17 expression vector | Invitrogen |
| MP13-23 | MP13-02, transformed with pDEST17-*bla*_OXA-48_ | (1) |
| MP13-24 | MP13-02, transformed with pDEST17-*bla*_OXA-48_-L67F | this study |
| MP13-25 | MP13-02, transformed with pDEST17-*bla*_OXA-48_-P68S | this study |
| MP13-26 | MP13-02, transformed with pDEST17-*bla*_OXA-48_-F72L | this study |
| MP13-27 | MP13-02, transformed with pDEST17-*bla*_OXA-48_-F156C | this study |
| MP13-28 | MP13-02, transformed with pDEST17-*bla*_OXA-48_-F156V | this study |
| MP13-29 | MP13-02, transformed with pDEST17-*bla*_OXA-48_-L158P | this study |
| MP13-30 | MP13-02, transformed with pDEST17-*bla*_OXA-48_-G160C | this study |
| MP13-31 | MP13-02, transformed with pDEST17-*bla*_OXA-48_-F72L/G131S | this study |
| MP13-32 | MP13-02, transformed with pDEST17-*bla*_OXA-48_-N146S/L158P | this study |
| ^a^ previously named as MP101(1) | | |

**References**

1. Fröhlich C, Sørum V, Thomassen AM, Johnsen PJ, Leiros HS, Samuelsen Ø. 2019. OXA-48-Mediated Ceftazidime-Avibactam Resistance Is Associated with Evolutionary Trade-Offs. mSphere 4.

2. Baba T, Ara T, Hasegawa M, Takai Y, Okumura Y, Baba M, Datsenko KA, Tomita M, Wanner BL, Mori H. 2006. Construction of *Escherichia coli* K-12 in-frame, single-gene knockout mutants: the Keio collection. Mol Syst Biol 2:2006 0008.
