# Supplementary material for: Cryptic β-Lactamase Evolution Is Driven by Low β-Lactam Concentrations
Source: mSphere. 2021 Apr 28;6(2):e00108-21. doi: 10.1128/mSphere.00108-21 (PMC8092134; doi:10.1128/mSphere.00108-21)
Supplement: TABLE S2 [file mSphere.00108-21-st002.docx]

| Name^a^ | | 5 ́-sequence-3 ́ | Reference |
| --- | --- | --- | --- |
| preOXA-48 | FP (A) | TATATTGCATTAAGCAAGGG | (1) |
|  | RP (B) | CACACAAATACGCGCTAACC |  |
| M13 | FP | GTAAAACGACGGCCAG | Thermo Fisher Scientific |
|  | RP | CAGGAAACAGCTATGAC |  |
| T7 | FP | TAATACGACTCACTATAGGG | Thermo Fisher Scientific |
|  | RP | GCTAGTTATTGCTCAGCGG |  |
| F67L | FP | GGGCGAACCAAGCATTTTTTCCCGCATCTACC | this study |
|  | RP | GGTAGATGCGGGAAAAAATGCTTGGTTCGCCC |  |
| P68S | FP | CGGGCGAACCAAGCATTTTTATCCGCATCTACC | this study |
|  | RP | GGTAGATGCGGA TAAAAATGCTTGGTTCGCCCG |  |
| F72L | FP | GCATTTTTACCCGCATCTACCTTGAAAATTCCCAATAGCTTGATCG | this study |
|  | RP | CGATCAAGCTATTGGGAATTTTCAAGGTAGATGCGGGTAAAAATGC |  |
| L158P | FP | GTAGACAGTTTCTGGCCCGACGGTGGTATTCG | this study |
|  | RP | CGAATACCACCGTCGGGCCAGAAACTGTCTAC |  |
| F156C | FP | GGGCAATGTAGACAGTTGTTGGCTCGACG | this study |
|  | RP | CGTCGAGCCAACAACTGTCTACATTGCCC |  |
| F156V | FP | GGGCAATGTAGACAGTGTCTGGCTCGACGG | this study |
|  | RP | CCGTCGAGCCAGACACTGTCTACATTGCCC |  |
| G160C | FP | CAGTTTCTGGCTCGACTGTGGTATTCGAATTTCGG | this study |
|  | RP | CCGAAATTCGAATACCACAGTCGAGCCAGAAACTG |  |
| G131S-out | FR | Agcgaggcacgtatgagcaag | this study |
|  | RP | AATTTGGCGGGCAAATTCTTG |  |
| N146S-out | FP | Gtgaggacatttcgggcaatg | this study |
|  | RP | TACCATAATCGAAAGCATGTAGC |  |
| OXA-48-pro-f | | TTGACACAGATATTTATGATATAATAACTGAGTAAGCTTAACATAAGGAGGAAAAACATATGCGTGTATTAGCCTTATCGG | (2) |
| cat-r | | GTAGCACCAGGCGTTTAAGG | this study |
| p15A46 | | TCGTATGGGGCTGACTTCAG | this study |

^a^FP= forward primer, RP reverse primer

**References**

1. Samuelsen Ø, Naseer U, Karah N, Lindemann PC, Kanestrom A, Leegaard TM, Sundsfjord A. 2013. Identification of *Enterobacteriaceae* isolates with OXA-48 and coproduction of OXA-181 and NDM-1 in Norway. J Antimicrob Chemother 68:1682-5.

2. Jensen PR, Hammer K. 1998. Artificial promoters for metabolic optimization. Biotechnol Bioeng 58:191-5.
