# Supplementary material for: Cryptic β-Lactamase Evolution Is Driven by Low β-Lactam Concentrations
Source: mSphere. 2021 Apr 28;6(2):e00108-21. doi: 10.1128/mSphere.00108-21 (PMC8092134; doi:10.1128/mSphere.00108-21)
Supplement: TABLE S3 [file mSphere.00108-21-st003.docx]

| OXA-48 variants | Ampicillin | Piperacillin | Ceftazidime | Cefepime | Imipenem | Meropenem |
| --- | --- | --- | --- | --- | --- | --- |
| wild-type | 1 | 1 | 100 | 100 | 25 | 25 |
| L67F | 10 | 100 | 500 | 500 | 50 | 50 |
| P68S | 10 | 100 | 500 | 500 | 50 | 50 |
| F72L | 10 | 100 | 1000 | 1000 | 50 | 50 |
| F156C | 10 | 100 | 500 | 500 | 50 | 100 |
| F156V | 100 | 100 | 500 | 500 | 50 | 100 |
| L158P | 100 | 100 | 500 | 500 | 50 | 50 |
| G160C | 10 | 100 | 500 | 500 | 50 | 50 |
| F72L/G131S | 100 | 100 | 500 | 500 | 50 | 50 |
| N146S/L158P | 100 | 100 | 500 | 500 | 50 | 100 |
