# Supplementary material for: Cryptic β-Lactamase Evolution Is Driven by Low β-Lactam Concentrations
Source: mSphere. 2021 Apr 28;6(2):e00108-21. doi: 10.1128/mSphere.00108-21 (PMC8092134; doi:10.1128/mSphere.00108-21)
Supplement: TABLE S4 [file mSphere.00108-21-st004.docx]

|  | OXA-48:L67F |
| --- | --- |
| PDB no. | 7ASS |
| Diffraction source | BL14.2, Bessy |
| Wavelength (Å), Temperature (°C) | 0.9184, -173 |
| Crystal-detector distance (mm) | 174.9 |
| Rotation range per image (°), total rotation range (°) | 0.10, 190 |
| Space group | P2_1_2_1_2_1_ |
| *a*, *b*, *c* (Å) | 88.56, 108.48, 125.42 |
| Resolution range (Å) | 50.00-1.91 (1.94-1.91) |
| No. of unique reflections | 94247 (4653) |
| Multiplicity | 7.1 (7.3) |
| Completeness (%) | 100 (99.9) |
| R_merge_ (%) | 16.5 (187.0) |
| R_pim_(%) | 9.9 (111.0) |
| Mean 〈 *I*/σ_(_*_I_*_)_〉 | 9.6 (1.1) |
| C _1/2_ | 0.997 (0.583) |
| Overall *B*-factor from Wilson plot (Å^2^) | 14.4 |
| Resolution range (Å) | 23.56-1.91 |
| Final R_work_ (%) | 18.9 |
| Final R_free_ (%) | 22.98 |
| Molecules in asymmetric unit | 4 |
| No. of non-H atoms (all protein chains) | 8896 |
| -Ions (Cl) | 5 |
| -Ligand (2 ceftazidime molecules) | 76 |
| -Water | 797 |
| R.m.s. deviations |  |
| -Bonds (Å) | 0.011 |
| -Angles (°) | 1.028 |
| Average B-factors (Å^2^) | 30.3 |
| - Protein chains A/B/C/D | 31.1/26.9/29.4/30.9 |
| -Ion (Cl) | 46.9 |
| -Ligand (2 ceftazidime molecules) | 55.5 |
| -Water | 35.5 |
| Ramachandran plot |  |
| Most favored (%) | 96.9 |
| Allowed (%) | 3.1 |
